# Population pharmacokinetic modeling of vedolizumab for graft‐versus‐host disease prophylaxis in adults with allogeneic hematopoietic stem cell transplant

**DOI:** 10.1002/prp2.1257

**Published:** 2024-09-04

**Authors:** Timothy Waterhouse, Kyle Baron, Westley Eure, Chunlin Chen, Nathanael L. Dirks, Johan Jansson, Mona Akbari, Shailly Mehrotra

**Affiliations:** ^1^ Metrum Research Group Tariffville Connecticut USA; ^2^ Takeda Pharmaceuticals Inc. Cambridge Massachusetts USA; ^3^ Present address: Bayer Pharmaceuticals Whippany New Jersey USA; ^4^ Present address: AbbVie Cambridge Massachusetts USA

**Keywords:** allogeneic hematopoietic stem cell transplantation, graft‐versus‐host disease (GvHD), population pharmacokinetics, prophylaxis, vedolizumab

## Abstract

We aimed to characterize the population pharmacokinetics (PK) of vedolizumab for acute graft‐versus‐host disease prophylaxis in adults undergoing allogeneic hematopoietic stem cell transplantation (allo‐HSCT) and assess potential clinically relevant covariates. Dosing, patient characteristics, and PK from a phase 1b, open‐label, dose‐finding study of vedolizumab 75 mg initial dose escalated to 300 mg and a phase 3 study of vedolizumab 300 mg in patients receiving allo‐HSCT were analyzed using a two‐compartment population PK model with linear elimination. Covariates included age, race, weight, sex, albumin, lymphocyte count, GvHD type, and concomitant medications. Weight, albumin, and lymphocyte count were time‐varying covariates. Model selection was driven by goodness‐of‐fit criteria, precision of parameter estimates, and visual predictive checks. In 193 patients undergoing allo‐HSCT, vedolizumab PK were well described by a two‐compartment, linear PK model. Using reference covariate values, final parameter estimates (95% confidence intervals [CI]) were: clearance, 0.148 (0.136, 0.162) L/day; central volume of distribution, 3.12 (3.03, 3.21) L; intercompartmental clearance, 0.500 (0.408, 0.612) L/day; and peripheral volume of distribution, 3.95 (3.52, 4.44) L. Weight and albumin were the most important predictors of vedolizumab PK, with clearance decreasing by ≈20% for low body weight/high albumin and increasing by ≈30% for high body weight/low albumin. There was an inverse relationship between vedolizumab clearance and age, but no detectable effect for lymphocyte count or GvHD type. Post hoc analyses did not detect any relationship between vedolizumab PK and concomitant medications. In summary, the covariates studied did not have a clinically meaningful effect on the PK of vedolizumab.

## Graphical abstract

Alignment between model‐simulated and observed vedolizumab concentration.

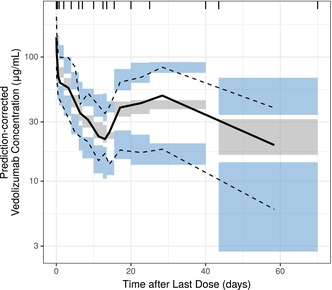

**Abbreviations**
%RSEs: percentage relative standard errors
aGvHD: acute graft‐versus‐host disease
AIC: Akaike information criterion
AUCss: area under the concentration‐time curve at steady state
AVA: anti‐vedolizumab antibodies
CI: confidence intervals
CL: clearance
CV%: coefficient of variation
DAMPs: damage‐associated molecular patterns
ECL: electrochemiluminescence
GI: gastrointestinal
GvHD: graft‐versus‐host disease
HSCT: allogeneic hematopoetic stem cell transplantation
IBD: inflammatory bowel disease
IgG1: immunoglobulin G1
IOV: inter‐occasion variability
Km: concentration at half‐maximum elimination rate
MAdCAM‐1: mucosal cell adhesion molecule‐1
NPDE: normalized prediction distribution errors
PAMPs: pathogen‐associated molecular patterns
PK: pharmacokinetics
Q: intercompartmental clearance
SE: standard error
Vc: central volume of distribution
Vmax: maximum elimination rate
Vp: peripheral volume of distribution
VPC: visual predictive checks

## 1 INTRODUCTION

Allogeneic hematopoietic stem cell transplantation (allo‐HSCT) is a potentially curative therapeutic option for patients with hematologic malignancies, but is frequently complicated by the emergence of graft‐versus‐host disease (GvHD).^1^, ^2^ GvHD is a major and potentially lethal complication of allo‐HSCT that results in considerable patient morbidity and mortality,^1^, ^2^ and displays a heterogeneous clinical presentation, with an acute form most commonly involving the skin, gastrointestinal (GI) tract and liver, and a chronic form involving other areas of the body.^1^, ^2^, ^3^

In acute GvHD, tissue damage due to the allo‐HSCT conditioning regimen leads to the release of damage‐associated molecular patterns (DAMPs) and pathogen‐associated molecular patterns (PAMPs) that cause activation of host antigen‐presenting cells.^4^ Donor T cells then become primed in the secondary lymphoid tissue of the host and subsequently migrate to target organs such as the GI tract where inflammatory cytokines and effector T cells damage the gut mucosa, causing the symptoms of acute GvHD.^5^, ^6^ The trafficking of T cells to GI mucosa and gut‐associated lymphoid tissue is mediated through adhesive interactions between integrin α4β7 and mucosal addressin cell adhesion molecule‐1 (MAdCAM‐1).^7^ Additionally, integrin α4β7 has been shown to be significantly increased on the surface of naive and memory T cells in individuals who subsequently developed intestinal acute GvHD (aGvHD) following allo‐HSCT, versus those who developed skin aGvHD or no GvHD.^5^, ^8^ Studies in murine models of aGvHD suggest that preventing T‐cell trafficking to gut‐associated lymphoid tissue through disrupting interactions between integrin α4β7 and MAdCAM‐1 may reduce or prevent the occurrence of aGvHD,^9^, ^10^, ^11^ indicating that integrin α4β7 represents a viable target for the treatment or prevention of aGvHD.

Vedolizumab (ENTYVIO®, Takeda Pharmaceuticals, Inc.) is a humanized immunoglobulin G1 (IgG1) monoclonal antibody that selectively targets integrin α4β7 and is used to treat patients with moderate to severe active ulcerative colitis or Crohn's disease.^12^, ^13^ Vedolizumab inhibits the interaction between integrin α4β7 and MAdCAM‐1, preventing the migration of gut‐homing leukocytes into the GI mucosa,^14^, ^15^ and provides gut‐selective, anti‐inflammatory effects without systemic immunosuppression.^16^

Studies have investigated vedolizumab for the treatment of aGvHD.^17^, ^18^, ^19^ Although a phase 2a clinical trial in a small number of patients with advanced, severe disease failed to demonstrate positive proof of concept for the efficacy of vedolizumab treatment in patients with steroid‐refractory GvHD,^17^ interest is growing in the use of vedolizumab for prophylaxis of intestinal aGvHD.^15^, ^20^, ^21^ A phase 1 trial has demonstrated that intravenous (IV) vedolizumab 300 mg was well tolerated when added to tacrolimus and methotrexate for GvHD prophylaxis, and patients receiving vedolizumab at this dose experienced a relatively low incidence of lower intestinal aGvHD.^15^ Additionally, a phase 3 clinical trial which evaluated the efficacy and safety of vedolizumab for the prophylaxis of intestinal aGvHD in patients undergoing allo‐HSCT (VEDO‐3035/GRAPHITE/NCT03657160), found that vedolizumab achieved a statistically significant and clinically meaningful improvement in lower gastrointestinal aGvHD‐free survival by Day 180 after allo‐HSCT.^17^

Previously, vedolizumab pharmacokinetics (PK) has been characterized in a population modeling study conducted in adults with ulcerative colitis or Crohn's disease.^22^ Vedolizumab PK was best described by a two‐compartment model with parallel linear and nonlinear clearance pathways.^22^ Extreme albumin and body weight values were found to have a potential to be clinically important predictors of clearance in patients with inflammatory bowel disease (IBD; effect sizes >25%).^22^ Patients with IBD and those requiring allo‐HSCT for malignant and nonmalignant hematologic conditions are different patient populations and the pathophysiology of IBD and hematologic conditions are heterogeneous.

In this study, a previously developed population PK model^22^ was refined to describe the disposition of vedolizumab administered for aGvHD prophylaxis in two clinical studies. We aimed to characterize the PK of IV vedolizumab for aGvHD prophylaxis in adults undergoing allo‐HSCT, and to assess the impact of potential clinically relevant covariates on vedolizumab PK in these populations.

## 2 METHODS

### 2.1 Study design and analysis populations

Data were derived from two previously reported clinical studies: VEDO‐1015, a phase 1b, open‐label, dose‐finding study of vedolizumab (NCT02728895) and VEDO‐3035 (GRAPHITE/NCT03657160); a phase 3 study to evaluate the efficacy of vedolizumab when added to background standard of care aGvHD prophylaxis in patients who received allo‐HSCT.^15^ Briefly, the studies were conducted in patients undergoing allo‐HSCT for a haematologic malignancy or myeloproliferative disorder (full inclusion and exclusion criteria have been published previously).^17^

#### 2.1.1 Phase 1b study: VEDO‐1015

The phase 1b study followed a rule‐based, dose‐finding study design with PK guidance.^15^ An initial dose‐finding phase was used to inform a subsequent expansion phase assessing the tolerability and clinical activity of vedolizumab.^15^

During the study, vedolizumab was administered at doses of 75 mg (three patients) or 300 mg (21 patients) as a 30‐min IV infusion on Day −1 (before allo‐HSCT) and on Days +13 and +42 (after allo‐HSCT).^15^ All participants received either a myeloablative or reduced‐intensity conditioning regimen, followed by tacrolimus and methotrexate (on Days +1, +3, +6, and +11 after allo‐HSCT).^15^ Serial blood samples were collected for PK analysis. Samples were taken on dosing days (Days −1 and +13 [pre‐dose, and 0.5, 2, 12, and 24 h post‐dose], on Day +42 [pre‐dose, and 0.5, 1, and 2 h post‐dose]), and on non‐dosing days (Days +3, +5, +7, +9, +11, +16, +18, +20, +22, +26, +30, +36, +40, and + 100; one sample taken per visit).^15^ The presence of anti‐vedolizumab antibodies (AVA) in serum was also evaluated, with samples taken on Days −1 (pre‐dose), 20, 34, and 100. An additional sample may have been collected from any patient who developed aGvHD.

#### 2.1.2 Phase 3 study: VEDO‐3035

This phase 3, randomized, double‐blind, placebo‐controlled, study evaluated vedolizumab 300 mg IV dose given as an infusion over 30 min or matched placebo at Days −1, +13, +41, +69, +97, +125, and +153 after allo‐HSCT in patients aged ≥18 years undergoing their first allo‐HSCT for the treatment of haematologic malignancy or myeloproliferative disorder. Serum samples for PK and AVA were taken on dosing days (Days −1, +13, +41, +69, +97, +125, and +153) as well as Day +180 and the end of treatment visit.

#### 2.1.3 Ethics approval statement

The studies were approved by each site's institutional review board, and all participants provided informed consent in accordance with the principles of the Declaration of Helsinki and the International Council for Harmonization Guidelines for Good Clinical Practice.

Nomenclature used for drug and molecular target conforms to the IUPHAR/BPS Guide to Pharmacology nomenclature classification.^23^

#### 2.1.4 Analytical assay

Serum concentrations of vedolizumab were determined using a sandwich enzyme‐linked immunosorbent assay method with a validated range of 0.20–8.0 μg/mL, as noted previously.^22^ The presence of AVA in serum was detected using validated electrochemiluminescence (ECL) assays.^24^

### 2.2 PK modeling

#### 2.2.1 Data management

In this analysis, data from the allo‐HSCT patients from the phase 1b study (VEDO‐1015) and the phase 3 study (VEDO‐3035) who were administered at least one dose of vedolizumab and for whom at least one quantifiable post‐dose plasma PK sample was available were included in the PK analysis. Dosing, PK, and covariate data from patients who received vedolizumab 75 and 300 mg^15^ were merged and assembled into NONMEM®‐ready dataset using R (version 4.1).^25^ Longitudinal vedolizumab serum concentration measurements were used for the PK analysis. Covariates included in the dataset were age, body weight, sex, serum albumin level, absolute lymphocyte count, evidence of aGvHD (liver, skin, or intestine), and anti‐vedolizumab status. Weight, albumin, absolute lymphocyte count, and evidence of aGvHD (at any time during the study and of any grade) were all included as time‐varying covariates, with interpolation of values between observed time points performed using a next observation carried backward approach. The remaining covariates were patient‐level or baseline factors.

#### 2.2.2 Population PK model development

The population PK analysis was performed using NONMEM® software (version 7.5, ICON Plc, Ireland). The first‐order conditional estimation with interaction method was used for model development.^26^

First, a base model was developed without considering covariate effects. The previously developed population PK model in patients with IBD was used as the starting point for the development of the base model in this analysis, after removal of all covariates.^22^ The structural form of the previous model was a two‐compartment model with zero‐order input and parallel linear and nonlinear clearance pathways, parameterized in terms of clearance (CL), central volume of distribution (*V*_c_), intercompartmental clearance (*Q*), peripheral volume of distribution (*V*_p_), maximum elimination rate (*V*_max_), and concentration at half‐maximum elimination rate (*K*_m_), with appropriate random effect distributions.^22^ Additional structural models were tested, including a standard two‐compartment linear PK model. Appropriate models for interindividual variability and residual error were also investigated during base model development. Interindividual variability was described using an exponential random effects model with the assumption of a log‐normal PK parameter distribution. For the residual error model, both proportional and combined additive and proportional models were tested. Inter‐occasion variability (IOV) was modeled on CL using an exponential random effect. A new occasion was defined whenever a vedolizumab dose was administered with subsequent vedolizumab PK observations taking place prior to the next administered dose. The assessment of model suitability and decisions about increasing or decreasing model complexity were guided both by goodness‐of‐fit criteria. Goodness‐of‐fit criteria included diagnostic scatter plots, successful convergence of the minimization routine, plausibility and precision of parameter estimates, correlation between model parameter estimation errors of <0.95 and Akaike information criterion (AIC). Measures of estimation precision included percentage relative standard errors (%RSEs) and 95% confidence intervals (CI) derived from NONMEM®‐reported SEs.

Second, a full covariate model emphasizing parameter estimation rather than stepwise hypothesis testing was implemented, owing to typical problems encountered when using stepwise regression techniques.^27^ The full covariate model was developed by incorporating the effect of all prespecified covariate–parameter relationships into the base model, while avoiding simultaneous inclusion of moderate to highly collinear/correlated covariates (absolute values of correlation coefficients >.30). Determination of the prespecified covariates was based on clinical interest and previous knowledge of the clinical pharmacological profile of vedolizumab.^22^ These covariate–parameter relationships included the effect of albumin on CL, the effect of body weight on CL, *V*_c_, *Q*, and *V*_p_, and the effect of absolute lymphocyte count on CL. The full covariate model was constructed by simultaneously including the effects of these prespecified covariates in the base model (Equation S1). The impact of body weight on vedolizumab PK was described using an allometric model with a reference weight of 75 kg (estimated and fixed allometric exponents were investigated). Other continuous covariates were included in the model as power functions normalized by a reference value. Categorical covariates were included in the model as power functions, with a dichotomous (0, 1) power coefficient serving as an on–off switch for each effect.

Inferences regarding the clinical relevance of each covariate from the full model were based on both the resulting parameter estimate and estimate precision. Uncertainty distributions of estimates for CL and the covariate parameters were constructed by simulating from a multivariate normal distribution derived from the NONMEM® covariance matrix of estimates. The median simulated CL estimate for the reference individual was designated as the “reference CL.” Distributions of the reference CL adjusted for a given covariate value were constructed on the basis of the median and 90% CI from the uncertainty distribution of the covariate–parameter estimates. The covariates were evaluated in a univariate fashion (i.e., covariates were fixed at their reference value except when perturbed). All derived CL values were normalized to the reference CL to reflect the relative change in CL versus the reference individual. The resulting distributions of relative CL for a given covariate value were visualized using a forest plot.

As a hypothesis‐generating exercise, an exploratory post hoc analysis was performed to investigate the potential impact of aGvHD on CL. Exploratory graphics included individual random effects for CL from the final model versus type of aGvHD (skin, intestinal, or liver). Additional post hoc analyses explored the influence of select concomitant medications on vedolizumab PK.

### 2.3 Model evaluation

Standard goodness–of‐fit diagnostic plots, simulation‐based visual predictive checks (VPC), and normalized prediction distribution errors (NPDE) were used to assess the predictive performance of the model. For the VPC, 500 Monte Carlo simulation replicates of the original dataset were generated using the final population PK model. Observed vedolizumab concentrations were plotted versus time after the first dose, including the observed median and 5th and 95th percentiles. Observed plots were then overlaid with the 95% CI of the median, 5th and 95th percentiles of the simulated data for comparison.

NPDE were plotted against population‐predicted vedolizumab concentrations and time after the first dose to check for evidence of model mis‐specification and were also plotted against covariates included in the model to gauge the presence of any bias. Histograms of the NPDE were then plotted to evaluate their distribution, which should be normal with a variance of 1 and a mean of 0.

## 3 RESULTS

### 3.1 Analysis population

Patients were 18–72 years of age; 57.0% were male and 43.0% female, and most patients were white (143 subjects, 74.1%; Table 1). Patients had a mean age of 50.8 years at baseline (Table 1). The final population PK dataset was comprised of data from 193 participants from two studies contributing a total of 2380 vedolizumab observations. Of these, 15 samples (0.6%) were below the limit of quantification (0.2 μg/mL) and were subsequently excluded from the analysis. In addition, four observations were excluded from the analysis because they were either below the limit of quantification immediately after the end of infusion or were unusually high concentrations. Six patients (3.1%) had evidence of liver GvHD at any time, while 62 (32.1%) had skin GvHD and 17 (8.8%) had intestinal GvHD. Two patients had antibodies against vedolizumab during the vedolizumab dosing period; positive tests for both patients occurred on the first day of dosing. At baseline, mean absolute lymphocyte count was 0.426 K/μL. Mean albumin for patients at baseline was within the normal range, at 3.97 g/dL.

**TABLE 1:** Covariates of study participants contributing vedolizumab observations to the pharmacokinetic dataset.

Covariates	Number of patients, *n* (%)		
	Study VEDO‐1015 (*n* = 24)	Study VEDO‐3035 (*n* = 169)	All data (*n* = 193)
Sex			
Male	7 (29.2)	103 (60.9)	110 (57.0)
Female	17 (70.8)	66 (39.1)	83 (43.0)
Race			
Asian	0 (0.0)	30 (17.8)	30 (15.5)
Black	1 (4.2)	3 (1.8)	4 (2.1)
White	22 (91.7)	121 (71.6)	143 (74.1)
Not reported	1 (4.2)	15 (8.9)	16 (8.3)
Type of GvHD^a^			
Liver	1 (4.2)	5 (3.0)	6 (3.1)
Intestine	3 (12.5)	14 (8.3)	17 (8.8)
Skin	14 (58.3)	48 (28.4)	62 (32.1)
Dose level (mg)			
75	3 (12.5)	0	3 (1.6)
300	21 (87.5)	169 (100.0)	190 (98.4)
Concomitant medication			
Methotrexate	19 (79.2)	129 (76.3)	148 (76.7)
Tacrolimus	24 (100.0)	84 (49.7)	108 (56.0)
Cyclosporine	0 (0.0)	85 (50.3)	85 (44.0)
Ursodeoxycholic acid	22 (91.7)	135 (79.9)	157 (81.3)

	Mean (SD)	Mean (SD)	Mean (SD)
Age (years)	49.9 (18.3)	50.9 (14.4)	50.8 (14.9)
Albumin (g/dL)	35.6 (4.16)	40.3 (5.54)	39.7 (5.60)
Lymphocytes (K/μL)	0.0697 (0.0687)	0.477 (0.776)	0.426 (0.739)
Baseline weight (kg)	90.3 (25.3)	76.8 (17.5)	78.5 (19.1)

Abbreviations: GvHD, graft‐versus‐host disease; SD, standard deviation.

^a^ Represents a summary of counts based on the type of GvHD reported at any time during the study. An occurrence of GvHD included any grade of GvHD (Grades I–IV), and a patient could exhibit one or more types of GvHD.

### 3.2 Population PK modeling results

#### 3.2.1 Base PK model

Initially, a two‐compartment model with linear and nonlinear clearance was evaluated on the basis of a previous population PK analysis of vedolizumab in patients with ulcerative colitis and Crohn's disease.^22^ However, the absence of adequate data at low concentrations (only three patients received the 75 mg dose) precluded adequate estimation of the nonlinear clearance parameters (*V*_max_ and *K*_m_ and model development continued with only linear clearance). A two‐compartment, linear population PK model continued to provide the best and most parsimonious fit to the data. The base model was implemented in NONMEM® with the ADVAN3 TRANS4 subroutine, and was parameterized in terms of CL, *V*_c_, *Q*, and *V*_p_. The base model also included fixed allometric weight effects on all clearance and volume parameters. Both models resulted in similar AIC values and goodness‐of‐fit diagnostics, indicating that the data were adequately described by the more parsimonious two‐compartment model with linear CL only (selected base model), compared with the two‐compartmental model with linear and nonlinear clearance.

**FIGURE 1:**
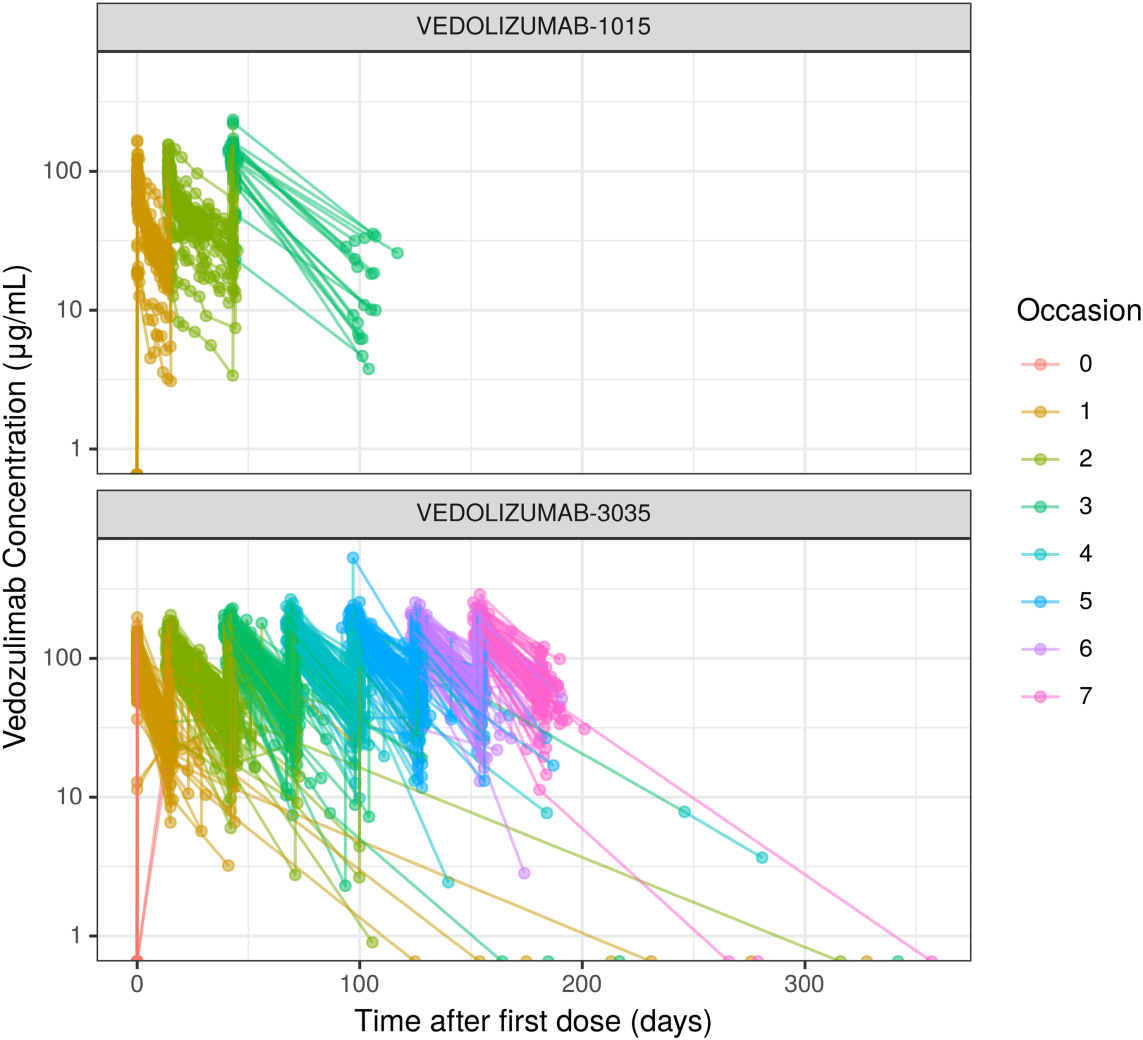
Composite plots of vedolizumab concentrations versus time after first dose in (A) VEDO‐1015 and (B) VEDO‐3035 (GRAPHITE). Occasion: A new occasion was defined whenever a vedolizumab dose was administered with subsequent vedolizumab PK observations taking place prior to the next administered dose.

Composite plots of vedolizumab concentrations over time after the first dose and time after the most recent dose are shown in Figure 1. Peak vedolizumab concentrations appear to increase with time over the first several occasions before reaching steady state. The parameter estimates for the base PK model are shown in Table S1. During model development, various random effects and residual error structures were tested. The final base model included IIV, using exponential variance models, on CL, *V*_c_, and *V*_p_ in a full block matrix: IIV percent coefficient of variation (CV%) was 44.0%, 22.5%, and 39.0%, respectively. The IOV was estimated on CL and the CV% was 21.5%. These estimates of random effects parameters show that clearance was moderately variable, both between individuals and between dosing occasions. The residual error model included only a proportional term (CV% = 15.9). All parameters were well estimated with adequate precision as judged by the CI ranges. Shrinkage was low for IIV of CL and *V*_c_ (8.31% and 6.40%, respectively), moderately high for IIV of *V*_p_ (26.4%), and high for IOV of CL (56.1%). The base model provided an adequate description of the data, aside from one particularly high observed concentration (above 500 μg/mL), the population and individual predicted vedolizumab concentrations matched the observations well. No systematic bias was noted in the conditional‐weighted residual or NPDE versus time, population predicted concentration, or time after dose plots. aside from a tendency toward low NPDE values for later times after dose, where data were sparse. Similarly, there was no systematic bias in NPDE versus covariate plots, except for some deviations for low weight and albumin values.

The random effects were mostly symmetric around zero, with positive correlations observed between random effects for CL and *V*_c_ and for *V*_c_ and *V*_p_, and little to no correlation between random effects for CL and *V*_p_ (Figure S1). There were some obvious trends evident in the relationships between interindividual random effects and continuous covariates: between body weight and CL, *V*_c_, and *V*_p_, and between albumin and CL. There were also some relationships observed between interindividual random effects and categorical covariates (study, sex, and race), although these are potentially explained by differences in body weight in these groups. There were no apparent trends between inter‐occasion random effects on CL and the time‐varying covariates, aside from a minor deviation for low albumin values, where data are sparse. Distributions of the IOV did not appear to vary over time. Plots of individual and population predictions of vedolizumab concentrations versus time matched the observed data well. The prediction‐corrected VPC for vedolizumab versus time after last dose showed a reasonable ability to predict the observed data overall. Diagnostic plots for the final base PK model are shown in Figures S2–S5.

#### 3.2.2 Final PK model (full model)

The final covariate model expanded the base model to include covariate effects. The final model included the effects of body weight on all clearance and volume terms, with estimated exponents for CL and *V*_c_ and fixed allometric exponents for *Q* (0.75) and *V*_p_ (1). Based on plots of random effects, the effect of albumin on CL was also included in the model. Effects of study, sex, and race were not included in the final model due to their association with bodyweight. The final model also included effects of age, lymphocyte count, and GvHD (liver, skin, and intestine) on CL, although there were no clear relationships observed between these covariates and random effects. The effect of antidrug antibodies on vedolizumab PK was not evaluated due to the limited number of subjects with positive ADA tests. Plots of individual model fits for the final vedolizumab PK model are provided in Figure S6.

**FIGURE 2:**
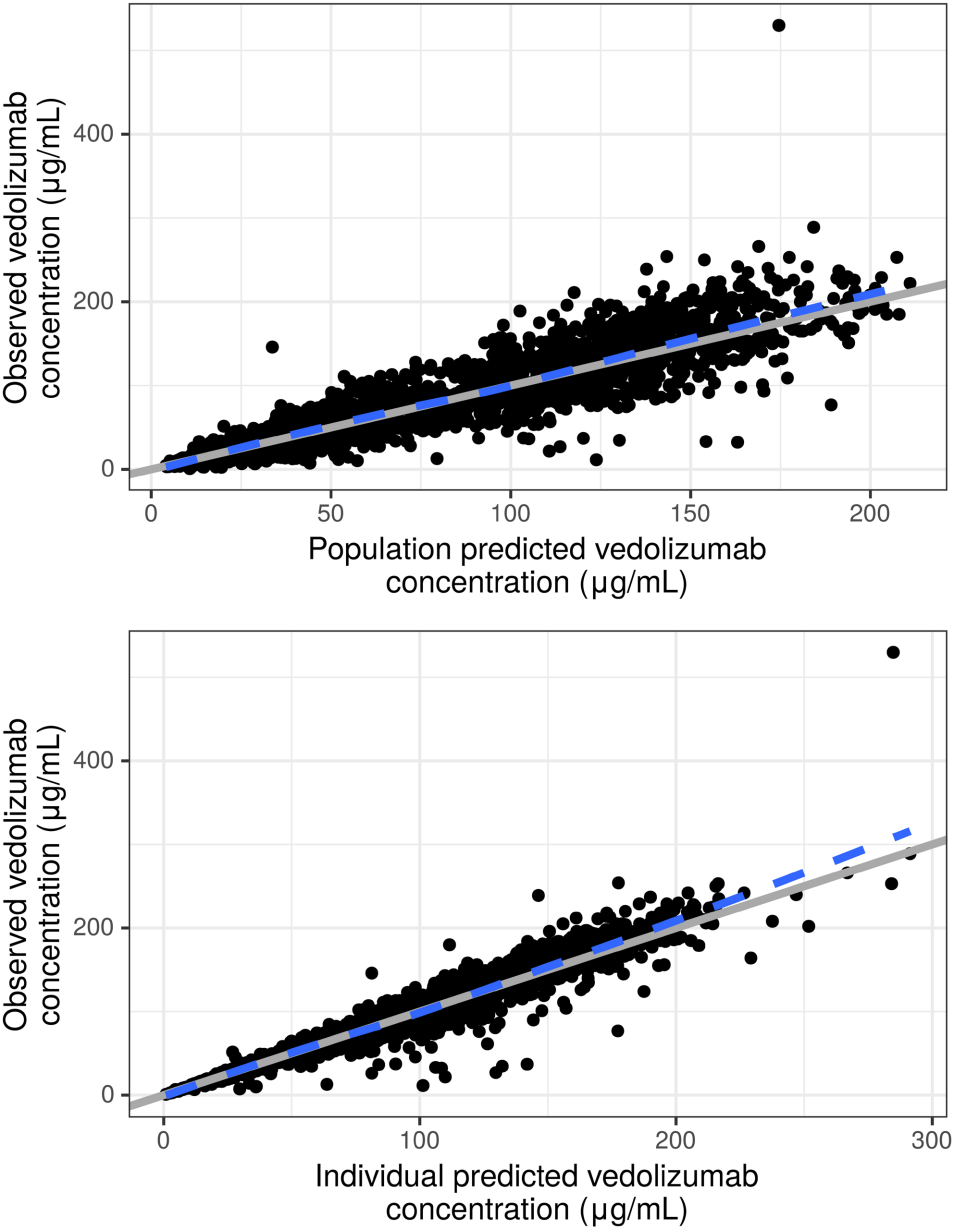
Observed versus population‐predicted (A) and individual‐predicted (B) vedolizumab concentrations. Observed values are indicated by black circles, the dashed blue line is a LOESSs smooth, and the line of identity (solid gray) is included as a reference.

In the final population PK model, parameter estimates were 0.148 L/day (CL), 3.12 L (*V*_c_), 3.95 L (*V*_p_), and 0.500 L/day (*Q*), using typical reference covariates for body weight (75 kg), albumin (4 g/dL), and absolute lymphocyte count (0.1 K/μL; Table 2, Equation S2). Final estimates of unexplained variability in CL, *V*_c_, and *V*_p_ were 29.4 percentage coefficient of variation (CV%), 18.1 CV%, and 44.2 CV%, respectively. Shrinkage values for the CL and *V*_c_, interindividual random effects were small with estimates of 12.3%, and 8.82%, respectively (estimates based on the standard deviation scale). IOV for CL was estimated at 17.9 CV% (Table 3). Most structural model parameters, covariate effects and random effect terms were estimated with reasonable precision based on %RSE and 95% CI results. Standard goodness‐of‐fit plots, including model predictions versus observations, residuals versus model predictions and residuals versus time, revealed that the final model was generally consistent with the observed data and minimal systematic bias remained (Figure 2 and Figure S7).

**TABLE 2:** Parameter estimates for the final vedolizumab model.

Parameters: Structural	Estimate	95% CI	RSE (%)	Units
CL	0.148	0.136–0.162	4.49	L/day
*V*_c_	3.12	3.03–3.21	1.44	L
*Q*	0.500	0.408–0.612	10.4	L/day
*V*_p_	3.95	3.52–4.44	5.93	L

Covariate effect parameters	Estimate	95% CI	RSE (%)
Body weight effect on CL	0.713	0.523, 0.902	13.5
Albumin effect on CL	−1.35	−1.67, −1.02	12.3
Lymphocyte count effect on CL	−0.0180	−0.0524, 0.0164	97.6
Age effect on CL	−0.130	−0.242, −0.0185	43.7
Body weight effect on *V*_c_	0.659	0.540, 0.778	9.21
Body weight effect on *V*_p_	1.00	Fixed	—
Body weight effect on *Q*	0.50	Fixed	—
Liver GvHD effect on CL	1.05	0.834, 1.26	10.3
Skin GvHD effect on CL	1.03	0.940, 1.12	4.56
Intestine GvHD effect on CL	1.07	0.870, 1.27	9.54

*Note*: Estimates are for a reference patient (age = 53 years, weight = 75 kg, albumin = 4 g/dL, lymphocytes = 0.1 K/μL, and no GvHD in liver, skin, or intestine). The 95% CI were derived using SEs from the NONMEM® $COVARIANCE step. CI = estimate ±1.96 × SE.

Abbreviations: CI, confidence intervals; CL, clearance; *Q*, intercompartmental clearance; RSE, relative standard error; SE, standard error; *V*_c_, central volume of distribution; *V*_p_, peripheral volume of distribution.

**TABLE 3:** Random effect parameter estimates for the final vedolizumab model.

Interindividual variance parameters	Estimate	95% CI	Shrinkage (%)
CL	0.0827 [CV% = 29.4]	0.0588, 0.107	12.3
*V*_c_	0.0323 [CV% = 18.1]	0.0235, 0.0411	8.82
*V*_p_	0.179 [CV% = 44.2]	0.0920, 0.265	25.3
Inter‐occasion variance parameters			
IOV‐CL	0.0315 [CV% = 17.9]	0.0190, 0.0440	55.4
Interindividual covariance parameters			
CL–*V*_c_	0.0214 [Corr = 0.415]	0.00903, 0.0338	—
CL–*V*_p_	−0.0253 [Corr = −0.208]	−0.0559, 0.00535	—
*V*_c_–*V*_p_	0.0272 [Corr = 0.358]	0.0101, 0.0443	—
Residual unexplained variance			
Proportional	0.0241 [CV% = 15.5]	0.0197, 0.0285	13.8

*Note*: Estimates are for a reference patient (age = 53 years, weight = 75 kg, albumin = 4 g/dL, lymphocytes = 0.1 K/μL, and no GvHD in liver, skin, or intestine). The 95% CI were derived using SEs from the NONMEM® $COVARIANCE step. CI = estimate ±1.96 × SE. CV% of log‐normal IIV = sqrt(exp(estimate)–1) × 100; where estimate is the variance estimate for inter‐individual variability. CV% of proportional residual error = sqrt(estimate) × 100; where estimate is the variance estimate for proportional error.

Abbreviations: CI, confidence intervals; CL, clearance; CV%, percentage coefficient of variation; IIV, inter‐individual variability; IOV, inter‐occasion variability; SE, standard error; *V*_c_, central volume of distribution; *V*_p_, peripheral volume of distribution.

#### 3.2.3 Final PK model (full model) covariate effects

**FIGURE 3:**
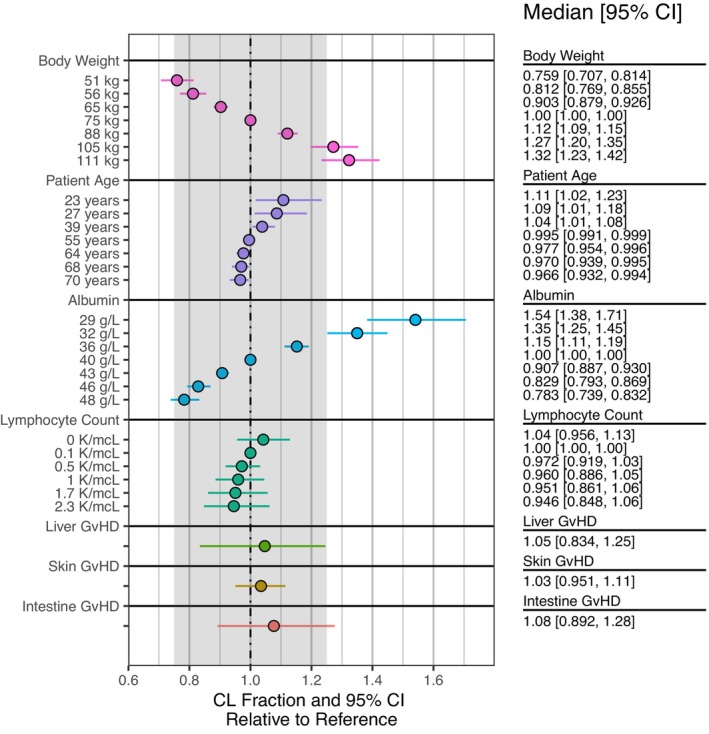
Covariate forest plot for relative vedolizumab clearance (CL) based on the final model. The colored circles represent the median and the solid horizontal lines represent the 95% confidence interval. Relative CL is shown for the 5th, 10th, 25th, 50th, 75th, 90th, and 95th percentiles of the observed values of body weight, age, albumin and lymphocyte count in the analysis dataset. A shaded region from −25% to +25% of the reference value has been added to provide visual context for each covariate effect. This shaded region should not be used to make statistical inferences about the magnitude of the covariate effects.

The relative changes in CL for a reference individual with various covariate values are illustrated in Figure 3. Median and 90% CI values for the reference individual reflect the uncertainty around the population estimate of CL for an individual with reference covariate values. For evaluation of covariates, the point marker and interval lines reflect uncertainty in the univariate covariate effect estimate and the change from the reference CL (i.e., the median posterior CL estimate for the reference individual).

Covariates that were identified as having potentially clinically relevant effects on CL included extreme values of albumin and body weight (Figure 3). Albumin was observed to be the most influential covariate and CL was observed to increase with decreasing albumin concentration. CL increased in line with increasing body weight, based on the allometrically scaled CL model (Figure 3). Following post hoc exploratory analysis, no clear relationships were evident between CL and type of aGvHD or concomitant medications after accounting for covariates included in the final population PK model (Figure S8).

#### 3.2.4 Final PK model evaluation

**FIGURE 4:**
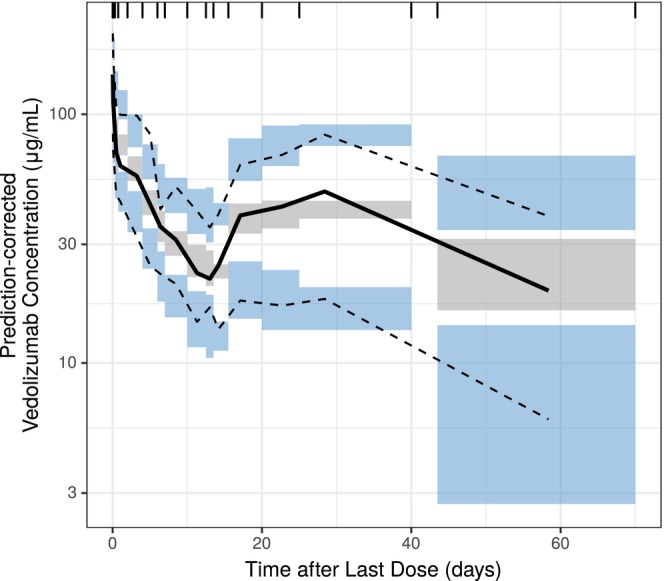
Final model: Prediction‐corrected visual predictive check of vedolizumab concentration versus time after most recent dose. Black lines represent the median (solid), 5th and 95th percentiles (dashed) of the observed data. Blue and gray‐shaded regions represent the 95% confidence intervals of the corresponding (i.e., 5th, 50th, and 95th) percentiles.

The VPC demonstrated good alignment between model‐simulated data and observed data (Figure 4), with the median, 5th and 95th percentiles of the observed data largely falling within the respective 95% prediction intervals of the simulated data. Evaluation of the NPDE indicated bias was neither present over population‐predicted vedolizumab concentrations or time after first dose (Figure S9), nor over the ranges of the covariates included in the final model (Figure S9).

## 4 DISCUSSION

This study is the first to assess population PK of vedolizumab in adult patients undergoing allo‐HSCT for GvHD prophylaxis and assess the effect of covariates on vedolizumab PK in this patient population. This analysis was based on pooled data from 24 patients from a previously reported phase 1b study, in which patients undergoing allo‐HSCT received vedolizumab 75 or 300 mg plus tacrolimus and methotrexate for GvHD prophylaxis^15^ and 169 patients from a phase 3 study to evaluate the efficacy of IV vedolizumab 300 mg when added to background GvHD prophylaxis in patients who received allo‐HSCT.

Using these data, vedolizumab PK were well described using a two‐compartment model with first‐order elimination, and the characteristics of vedolizumab PK were generally consistent with other therapeutic monoclonal antibodies of the IgG1 subclass.^28^ Additionally, PK disposition and identified significant covariates were consistent with the previous population PK analysis of vedolizumab performed in patients with IBD.^22^ In the evaluation of patients with IBD, the structural PK vedolizumab model was two‐compartment with zero‐order input and parallel linear and nonlinear clearance pathways, and was largely consistent with observations from this study, though only linear clearance was selected for use in the current final model.^22^ In patients with IBD, vedolizumab exposures decreased in a biexponential fashion after reaching peak concentrations, with a biexponential decrease observed down to approximately 1–10 ng/mL, after which concentrations appeared to decrease in a nonlinear fashion. The contribution of nonlinear elimination in IBD patients was, however, minimal to the total clearance of vedolizumab at the approved therapeutic dosage of 300 mg.^22^ In this analysis, vedolizumab exposure was also observed to decrease biexponentially, though attempted modeling of the nonlinear clearance component caused the structural model to collapse to a linear clearance (first‐order)‐only model. The parameters *V*_max_ and *K*_m_ were therefore not able to be estimated well in the absence of data at low concentrations. It is possible that nonlinear clearance of vedolizumab occurs in an allo‐HSCT population; however, these data were unable to support this, potentially because the majority of observed vedolizumab concentrations were >10 ng/mL, and limited PK data were available for the lower vedolizumab dose of 75 mg owing to the small number of patients in this group (*n* = 3). Importantly, the final PK model adequately described the observed data as judged by VPCs, NPDEs, and standard goodness‐of‐fit plots.

Covariates investigated in this study were chosen on the basis of previous modeling in patients with IBD and factors of specific clinical interest in populations undergoing allo‐HSCT (such as lymphocytes).^22^ Linear CL was found to be 0.159 L/day in patients with ulcerative colitis and 0.155 L/day for those with Crohn's disease, similar to that observed in the current population undergoing allo‐HSCT (0.148 L/day).^22^ In addition, relative similarities were also observed among the other PK parameters between the previous and current analyses, such as central volume of distribution with values of 3.19 and 3.12 L estimated for *V*_c_ ^22^; however, minor differences were observed between PK parameters of *V*_p_ and *Q*, which were higher in the GvHD model compared to the IBD population with values of 1.65 and 3.95 L estimated for *V*_p_; and 0.12 and 0.5 L/day estimated for *Q*, respectively. Larger volumes of vedolizumab partitioning to the peripheral compartments could potentially account for the higher steady‐state concentrations observed in the GvHD population compared to the IBD population. This study identified that extreme values of albumin and body weight may have impact on CL as also observed in the earlier study of patients with IBD.^22^

Final model parameter estimates and measures of estimation precision (95% confidence interval [CI]) were used to infer the clinical relevance of covariate effects based on a reference covariate group. In line with the previous population PK model in ulcerative colitis and Crohn's disease, a covariate effect size on vedolizumab clearance >25% was considered clinically meaningful.^22^

The clinical implications of the findings of this study for vedolizumab in a population undergoing allo‐HSCT must be considered. Notably, when all other conditions were held constant at the reference values (53‐year‐old patient weighing 75 kg with no GvHD in the liver, skin, or intestine, an albumin value of 40 g/L, and a lymphocyte count of 0.1 K/μL), vedolizumab AUC_ss_ increased by about 20% for subjects with low body weight (10th percentile: 51 kg) or high albumin (90th percentile: 46 g/L), and AUC_ss_ decreased by about 25% for subjects with high body weight or low albumin. At observed body weights ≥105 kg (90th percentile) or albumin concentrations 36 g/L (25th percentile), these covariates had the potential to be clinically important, based on effect sizes and 90% CIs resulting in a change in CL of >25%. Absolute lymphocyte count, age, and the presence and type of aGvHD had no clinically meaningful impact on CL, though this was anticipated because there were few participants representing each aGvHD subtype, which likely precluded full evaluation of this covariate effect. Additionally, only two subjects in the phase 3 study had antibodies against vedolizumab during the vedolizumab dosing period, though this may primarily be due to the administration of myeloablative therapy and immunosuppressant treatment following the first dose of vedolizumab. The scarcity of patients with AVA during the trial meant that the presence of AVA could not be evaluated as a covariate in this study. The findings of this study suggest that albumin level and body weight are statistically significant covariates that affect clearance, as observed in patients with IBD, though these parameters are likely to be relevant only at extreme values.^22^ The post hoc exploration of effects of concomitant medications on vedolizumab PK revealed no relationships between vedolizumab disposition and methotrexate, tacrolimus, cyclosporine, and ursodeoxycholic acid. Importantly, this study has illustrated that the population nonlinear mixed effects modeling approach has the potential to explore and identify other covariates of interest in populations undergoing allo‐HSCT not included in the current analysis (e.g., C‐reactive protein or inflammatory cytokines) which may act as predictors of disease emergence, response to treatment, and prognosis in aGvHD, further informing clinical practice.^29^, ^30^, ^31^

The strengths of this study were that a model was developed that adequately characterized the PK of vedolizumab in an allo‐HSCT population, as determined by standard goodness‐of‐fit criteria and model evaluation. This analysis was facilitated by the extensive PK sampling and informativeness of the sampling schedule for the original phase 1b study and the larger phase 3 study.^15^ However, study limitations must also be considered. Primarily, the small number of patients in each covariate group limits the conclusions that may be made about the covariate–parameter relationships of vedolizumab in patients undergoing allo‐HSCT for a hematologic malignancy and owing to the low number of patients in certain subgroups, conclusions could not be drawn regarding the effects of certain covariates (e.g., AVA and aGVHD). Similarly, differences in PK parameters between patients with hematologic malignancy versus those with myeloproliferative disorders could not be assessed due to the small number of patients with myeloproliferative disorders.

In conclusion, the PK of vedolizumab for GvHD prophylaxis in individuals undergoing allo‐HSCT were well described by a two‐compartment pharmacokinetic model with linear CL. Differences in the CL of vedolizumab relative to a typical reference individual were observed for those with extreme albumin levels and body weight. No clinically meaningful impacts on CL were observed for absolute lymphocyte count, and neither was an impact on CL observed for the presence or type of aGvHD or age. This study provides an understanding of vedolizumab PK in patients undergoing allo‐HSCT, which is broadly similar to the PK in patients with IBD, although we observed slightly higher steady‐state concentrations in the aGvHD population compared with IBD patients at the same dosing regimen (Q4W). The population PK model in this study can be used to conduct PK simulation‐based analyses to help inform dosage decisions of vedolizumab in future clinical trials with GvHD prophylaxis patients.

### AUTHOR CONTRIBUTIONS

All authors contributed to the design and conduct of the study, analysis and interpretation of the data, and review and approval of the final manuscript for submission.

## FUNDING INFORMATION

This study was sponsored by Takeda Pharmaceuticals.

## CONFLICT OF INTEREST STATEMENT

K. B. is an employee of Metrum Research Group, Tariffville, Connecticut. W.E. is an employee of Takeda Pharmaceuticals and receives stock or stock options. T.W. is an employee of Metrum Research Group, Tariffville, Connecticut. C.C. was an employee of Takeda Pharmaceuticals and received stock or stock options at the time of the study. N.L.D. is an employee of Metrum Research Group, Tariffville, Connecticut. J.J. is an employee of Takeda Pharmaceuticals and receives stock or stock options. M.A. was an employee of Takeda Pharmaceuticals and received stock or stock options at the time of the study, and is currently an employee of AbbVie Inc., Cambridge, Massachusetts. S.M. is an employee of Takeda Pharmaceuticals and receives stock or stock options.

## Supporting information


Data S1.

## Data Availability

The datasets, including the redacted study protocol, redacted statistical analysis plan, and individual participants data supporting the results reported in this article, will be made available within 3 months from initial request to researchers who provide a methodologically sound proposal. The data will be provided after its de‐identification, in compliance with applicable privacy laws, data protection, and requirements for consent and anonymization.

## References

[1] Ferrara JL , Levine JE , Reddy P , et al. Graft‐versus‐host disease. Lancet. 2009;373(9674):1550‐1561. 19282026 10.1016/S0140-6736(09)60237-3 PMC2735047

[2] Garnett C , Apperley JF , Pavlů J . Treatment and management of graft‐versus‐host disease: improving response and survival. Ther Adv Hematol. 2013;4(6):366‐378. 24319572 10.1177/2040620713489842 PMC3854558

[3] Billingham R . The biology of graft‐versus‐host reactions. Harvey Lect. 1966;62:21. 4875305

[4] Malard F , Holler E , Sandmaier BM , Huang H , Mohty M . Acute graft‐versus‐host disease. Nat Rev Dis Primers. 2023;9(1):27. doi:10.1038/s41572-023-00438-1 37291149

[5] Chen Y‐B , Kim HT , McDonough S , et al. Up‐regulation of α4β7 integrin on peripheral T cell subsets correlates with the development of acute intestinal graft‐versus‐host disease following allogeneic stem cell transplantation. Biol Blood Marrow Transplant. 2009;15(9):1066‐1076. 19660719 10.1016/j.bbmt.2009.05.003 PMC2945817

[6] Ghimire S , Weber D , Mavin E , Wang X , Dickinson AM , Holler E . Pathophysiology of GvHD and other HSCT‐related major complications. Front Immunol. 2017;8:79. 28373870 10.3389/fimmu.2017.00079 PMC5357769

[7] Hamann A , Andrew DP , Jablonski‐Westrich D , Holzmann B , Butcher EC . Role of alpha 4‐integrins in lymphocyte homing to mucosal tissues in vivo. J Immunol. 1994;152(7):3282‐3293. 7511642

[8] Khandelwal P , Davies SM , Jordan MB , et al. α4β7 integrin is upregulated on CD8+ effector memory T‐cells in children with gut GvHD prior to clinical symptoms and represents a therapeutic target in pediatric allogeneic HSCT patients. Biol Blood Marrow Transplant. 2019;25(3):S265‐S266.

[9] Murai M , Yoneyama H , Ezaki T , et al. Peyer's patch is the essential site in initiating murine acute and lethal graft‐versus‐host reaction. Nat Immunol. 2003;4(2):154‐160. 12524535 10.1038/ni879

[10] Waldman E , Lu SX , Hubbard VM , et al. Absence of β7 integrin results in less graft‐versus‐host disease because of decreased homing of alloreactive T cells to intestine. Blood. 2006;107(4):1703‐1711. 16291587 10.1182/blood-2005-08-3445 PMC1895413

[11] Ueha S , Murai M , Yoneyama H , et al. Intervention of MAdCAM‐1 or fractalkine alleviates graft‐versus‐host reaction associated intestinal injury while preserving graft‐versus‐tumor effects. J Leukoc Biol. 2007;81(1):176‐185. 17053165 10.1189/jlb.0306231

[12] US FaDA . Vedolizumab prescribing information. Accessed August 2020. https://wwwaccessdatafdagov/drugsatfda_docs/label/2014/125476s000lblpdf

[13] European MA . Vedolizumab summary of product characteristics. Accessed August 2020. https://wwwemaeuropaeu/en/documents/product‐information/entyvio‐epar‐product‐information_enpdf

[14] Salmi M , Jalkanen S . Lymphocyte homing to the gut: attraction, adhesion, and commitment. Immunol Rev. 2005;206(1):100‐113. 16048544 10.1111/j.0105-2896.2005.00285.x

[15] Chen Y‐B , Shah NN , Renteria AS , et al. Vedolizumab for prevention of graft‐versus‐host disease after allogeneic hematopoietic stem cell transplantation. Blood Adv. 2019;3(23):4136‐4146. 31821456 10.1182/bloodadvances.2019000893 PMC6963235

[16] Soler D , Chapman T , Yang L‐L , Wyant T , Egan R , Fedyk ER . The binding specificity and selective antagonism of vedolizumab, an anti‐α4β7 integrin therapeutic antibody in development for inflammatory bowel diseases. J Pharmacol Exp Ther. 2009;330(3):864‐875. 19509315 10.1124/jpet.109.153973

[17] Chen YB , Mohty M , Zeiser R , et al. Vedolizumab for the prevention of intestinal acute GVHD after allogeneic hematopoietic stem cell transplantation: a randomized phase 3 trial. Nat Med. 2024. doi:10.1038/s41591-024-03016-4. Epub ahead of print. PMC11333288 38844797

[18] Danylesko I , Bukauskas A , Paulson M , et al. Anti‐α4β7 integrin monoclonal antibody (vedolizumab) for the treatment of steroid‐resistant severe intestinal acute graft‐versus‐host disease. Bone Marrow Transplant. 2019;54(7):987‐993. 30356163 10.1038/s41409-018-0364-5

[19] Fløisand Y , Schroeder MA , Chevallier P , et al. A phase 2a randomized clinical trial of intravenous vedolizumab for the treatment of steroid‐refractory intestinal acute graft‐versus‐host disease. Bone Marrow Transplant. 2021;56:1‐12. 34108672 10.1038/s41409-021-01356-0 PMC8486663

[20] Palaniyandi S , Kumari R , Strattan E , et al. α4β7 blockade with vedolizumab is effective in prevention and treatment of experimental acute graft versus host disease. Biol Blood Marrow Transplant. 2019;25(3):S300‐S301.

[21] DiMaggio E . Acute graft versus host disease: emerging insights and updates into detection, prevention, and treatment. Pharmacotherapy. 2020;40(8):788‐807. 32530080 10.1002/phar.2436

[22] Rosario M , Dirks N , Gastonguay M , et al. Population pharmacokinetics‐pharmacodynamics of vedolizumab in patients with ulcerative colitis and Crohn's disease. Aliment Pharmacol Ther. 2015;42(2):188‐202. 25996351 10.1111/apt.13243 PMC5032981

[23] Alexander SP , Kelly E , Marrion NV , et al. The concise guide to pharmacology 2017/18: overview. Br J Pharmacol. 2017;174(Suppl 1):S1‐S16. doi:10.1111/bph.13882 29055037 PMC5650665

[24] Wyant T , Yang L , Rosario M . Comparison of the ELISA and ECL assay for vedolizumab anti‐drug antibodies: assessing the impact on pharmacokinetics and safety outcomes of the phase 3 GEMINI trials. AAPS J. 2021;23(1):1‐10. 10.1208/s12248-020-00518-0 PMC7669784 33200296

[25] RCT . The R project for statistical computing. Accessed October 2020. https://wwwr‐projectorg/

[26] Beal S , Sheiner L , Boeckmann A . NONMEM users guide—part VII. NONMEM Project Group; 1992.

[27] Gastonguay MR . Full covariate models as an alternative to methods relying on statistical significance for inferences about covariate effects: a review of methodology and 42 case studies (Abtract number 2229). Annual Meeting of the Population Approach Group in Europe Athens, Greece 2011 (June 7).

[28] Dirks NL , Meibohm B . Population pharmacokinetics of therapeutic monoclonal antibodies. Clin Pharmacokinet. 2010;49(10):633‐659. 20818831 10.2165/11535960-000000000-00000

[29] Minculescu L , Kornblit BT , Friis LS , et al. C‐reactive protein levels at diagnosis of acute graft‐versus‐host disease predict steroid‐refractory disease, treatment‐related mortality, and overall survival after allogeneic hematopoietic stem cell transplantation. Biol Blood Marrow Transplant. 2018;24(3):600‐607. 29074374 10.1016/j.bbmt.2017.10.025

[30] Fuji S , Kim S‐W , Fukuda T , et al. Preengraftment serum C‐reactive protein (CRP) value may predict acute graft‐versus‐host disease and nonrelapse mortality after allogeneic hematopoietic stem cell transplantation. Biol Blood Marrow Transplant. 2008;14(5):510‐517. 18410893 10.1016/j.bbmt.2008.02.008

[31] Chung H , Jang JE , Kim S‐J , Kim JS , Min YH , Cheong JW . Serum albumin and C‐reactive protein as significant predictors of non‐relapse mortality in lower gastrointestinal graft‐versus‐host disease. Ann Hematol. 2020;99:1111‐1119. 32253453 10.1007/s00277-020-04015-4

