# Supplementary material for: Population pharmacokinetic modeling of vedolizumab for graft‐versus‐host disease prophylaxis in adults with allogeneic hematopoietic stem cell transplant
Source: Pharmacol Res Perspect. 2024 Sep 4;12(5):e1257. doi: 10.1002/prp2.1257 (PMC11374527; doi:10.1002/prp2.1257)
Supplement: Supplementary file 1 — Data S1. [file PRP2-12-e1257-s001.docx]

# Supplementary Equations

# Equation S1. Effects of covariates.

Physiologic relationships were incorporated into the covariate-parameter models as appropriate. For example, the change in physiologic parameters as a function of body size was both theoretically and empirically described by an allometric model, described in the following equation:

Where:

- *P_ri_* denotes the typical value of the *r*th parameter in the *i*th individual, described as a function of individual body weight (WT*_i_*), normalized by a reference weight (ref_WT_).

In those cases where no physiologic relationship was known a priori, the effects of continuous covariates were modeled using a normalized power model (on the log scale) while the effects of categorical covariates were described by an exponential model:

Where:

- The typical value of a model parameter (*P_ri_)* is a function of *L* covariates, with *l* = 1,…, *M* representing the continuous covariates and *l* = *M*+1,…., *L* representing the binary categorical covariates, (cov*_il_*) in the *i*th individual.
- *θ_r_* represents is the typical value (i.e., population mean) the *r* th parameter for a subject with reference covariate values.
- *β_lr_* represents the effect of covariate *l* on pharmacokinetic parameter *r*.

For parameters that include both IIV and covariate effects, the above equations were combined to give:

# Equation S2. Final model equations.


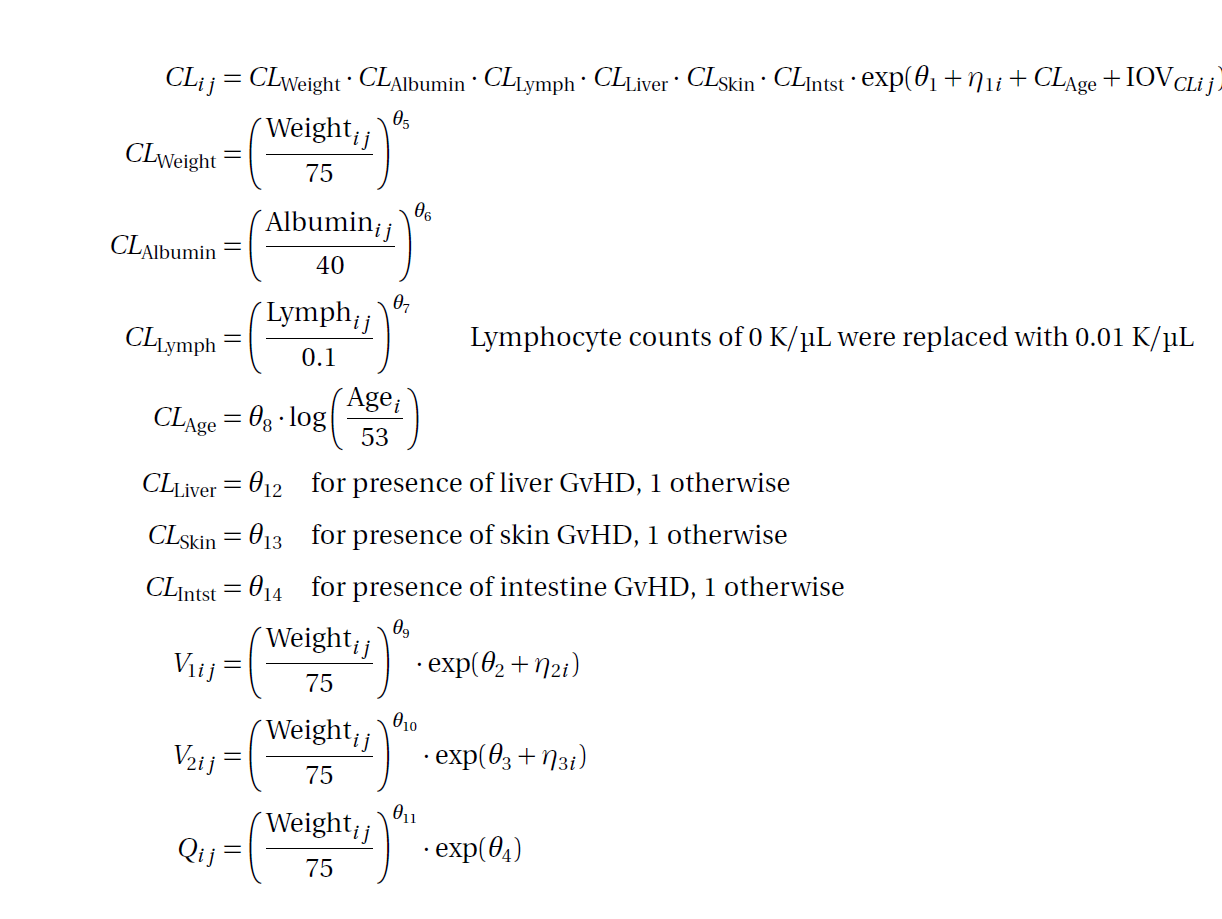


Where:

- for the *i*th individual at the *j*th time, the value for CL was described as a function of individual body weight, albumin, lymphocyte count, age, and GvHD (liver, skin, or intestine). Continuous covariates normalized to typical reference values included weight (reference 75 kg, exponent θ_5_), albumin (reference 40 g/L, exponent θ_6_), lymphocyte count (reference 0.1 K/μL, exponent θ_7_), which was further treated by replacing any zeroes with 0.01 K/μL, and age (reference 53 years, coefficient θ_8_). Effects of categorical covariates were estimated only for the non-reference categories, as the reference categories were numerically cancelled by an exponent value of 0 in the model; thus, covariate effects were estimated for liver, skin, and intestine GvHD (θ_11_, θ_12_, and θ_13_, respectively).
- the individual parameter values for the anatomical volume terms (V_1_ and V_2_) and Q were described as a function of individual bodyweight, normalized by the population reference weight, i.e., 75 kg, with power parameters of θ_9_ and θ_10_ for V_c_ and V_p_, respectively (fixed to 1 for V_p_), and θ_11_ for Q (fixed to 0.75).
- η_1i_ , η_2i_ , and η_3i_ are the estimates of interindividual variability of CL, V_c_ and V_p_, respectively, for the *i* th individual.
- IOV_CLi j_ is the estimate of interoccasion variability of CL for the *i* th individual at the *j* th time.

# Supplementary Tables

**Table S1** Parameter estimates for the base vedolizumab model

| **Parameter** | **Estimate** | **95% CI** | **RSE (%)** | **Units** |
| --- | --- | --- | --- | --- |
| CL | 0.149 | 0.138, 0.160 | 3.63 | L/day |
| V_c_ | 3.16 | 3.06, 3.27 | 1.72 | L |
| V_p_ | 5.03 | 4.63, 5.47 | 4.27 | L |
| Q | 0.585 | 0.504, 0.679 | 7.62 | L/day |
| **Inter-individual variability** | | | | |
| IIV-CL | 0.177 (CV%=44.0) | 0.131, 0.222 | 13.1 | variance |
| IIV-V_c_ | 0.0492 (CV%=22.5) | 0.0371, 0.0613 | 12.6 | variance |
| IOV-CL | 0.0453 (CV%=21.5) | 0.0218, 0.0688 | 26.5 | variance |
| Correlation CL-Vc | 0.0545 (Corr = 0.585) | 0.0359, 0.0732 | - | covariance |
| **Residual error** | | | | |
| Proportional | 0.0254 (CV%=15.9) | 0.0208, 0.0300 | 9.21 | variance |

NaN indicates that the lower limit is <0. The 95% CI were derived using standard errors from the NONMEM^®^ $COVARIANCE step. CI = estimate ± 1.96 × SE. CV% of log-normal IIV = sqrt(exp(estimate) – 1) × 100; where estimate is the variance estimate for inter-individual variability. CV% of proportional residual error = sqrt(estimate) × 100; where estimate is the variance estimate for proportional error.

CI, confidence intervals; CL, clearance; CV%, percentage coefficient of variation; IIV, inter-individual variability; IOV, inter-occasion variability; Q, intercompartmental clearance; RSE, relative standard error; SE, standard error; V_c_, central volume of distribution; V_p_, peripheral volume of distribution

# Supplementary Figures

**Figure S1.** Correlation and distribution of individual estimates of interindividual variability for vedolizumab.

The lower off-diagonal shows a bivariate scatter plot with values indicated by black circles and a dashed blue LOESS smooth trend line through the data. The diagonal shows density frequency (count) plots of the data, and the correlation coefficient is reported in the upper off-diagonal. CL, clearance; V1, central volume of distribution (V_c_); V2, peripheral volume of distribution (V_p_).

**Figure S2.** Observations versus population and individual predictions for the final base model.

Values are indicated by black circles, the solid grey line at x = y is a reference line, and the dashed blue line is a LOESS smooth trend line through the data.

**Figure S3.** Normalized prediction distribution errors versus population predictions, time and time after dose for the final base model.

Values are indicated by black circles, the solid grey line (residuals = -2, 0, 2) is a reference line, and the dashed blue line is a LOESS smooth trend line through the data.

# Figure S4. Conditional weighted residual versus population predictions, time and time after first dose for the final base model.

Values are indicated by black circles, the solid grey line (residuals = -2, 0, 2) is a reference line, and the dashed blue line is a LOESS smooth trend line through the data.

**Figure S5.** Prediction-corrected visual predictive check of vedolizumab concentration versus time after most recent dose for the final base model.

Black lines represent the median (solid) and 5^th^ and 95^th^ percentiles (dashed). Blue and grey shaded regions represent the 95% confidence intervals of the corresponding (i.e., 5^th^, 50^th^, and 95^th^) percentiles.

**Figure S6.** Individual plots of DV, PRED, and IPRED vs. TAFD for the final vedolizumab model. Observed values (DV) are shown as black points, population predicted values (PRED) are shown as a blue line, and individual predicted values (IPRED) are shown as a red line. PK data is plotted against time after first dose (TAFD). The subject ID and the vedolizumab dose the subject received in the study are shown in each plot header.


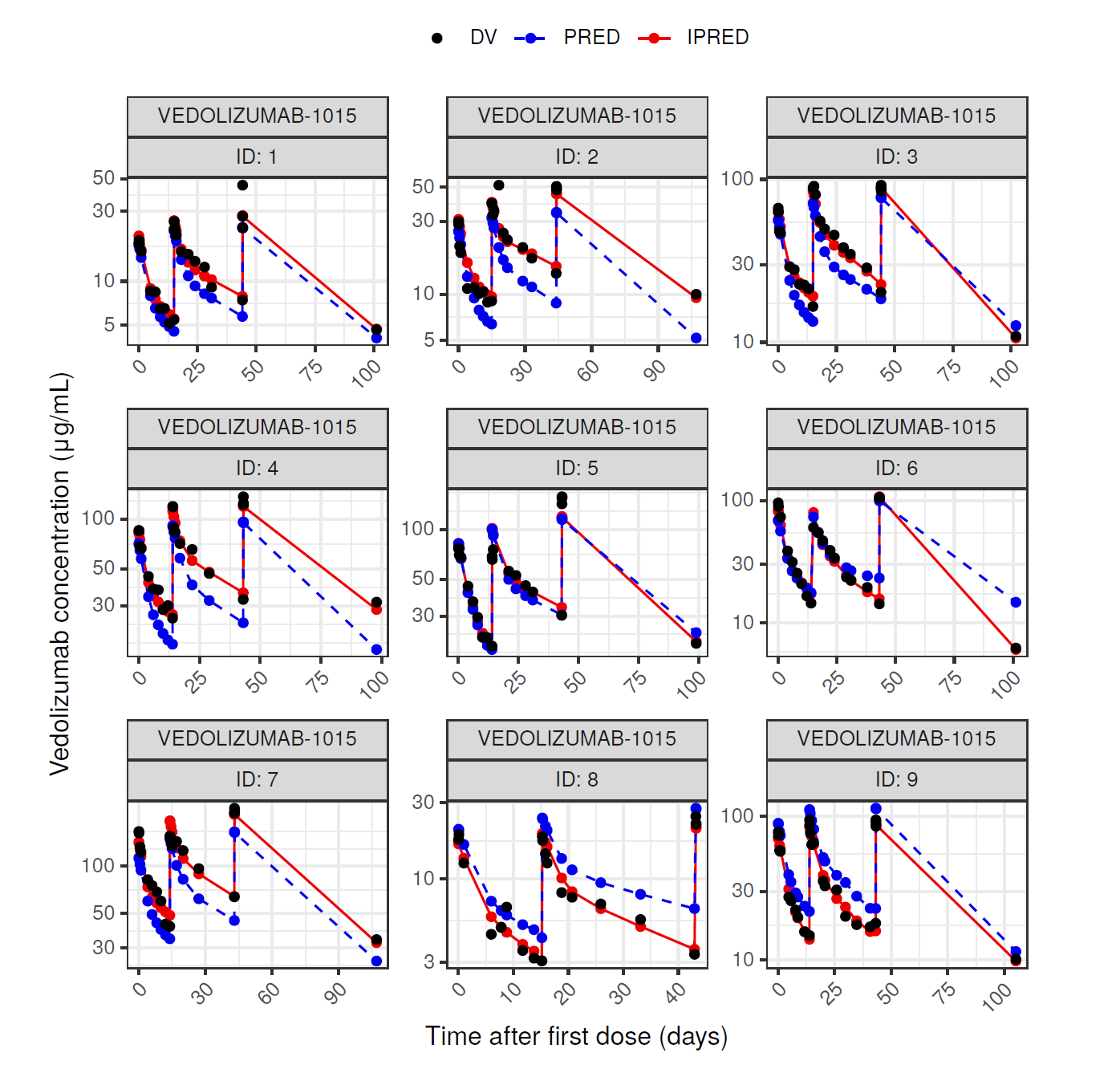


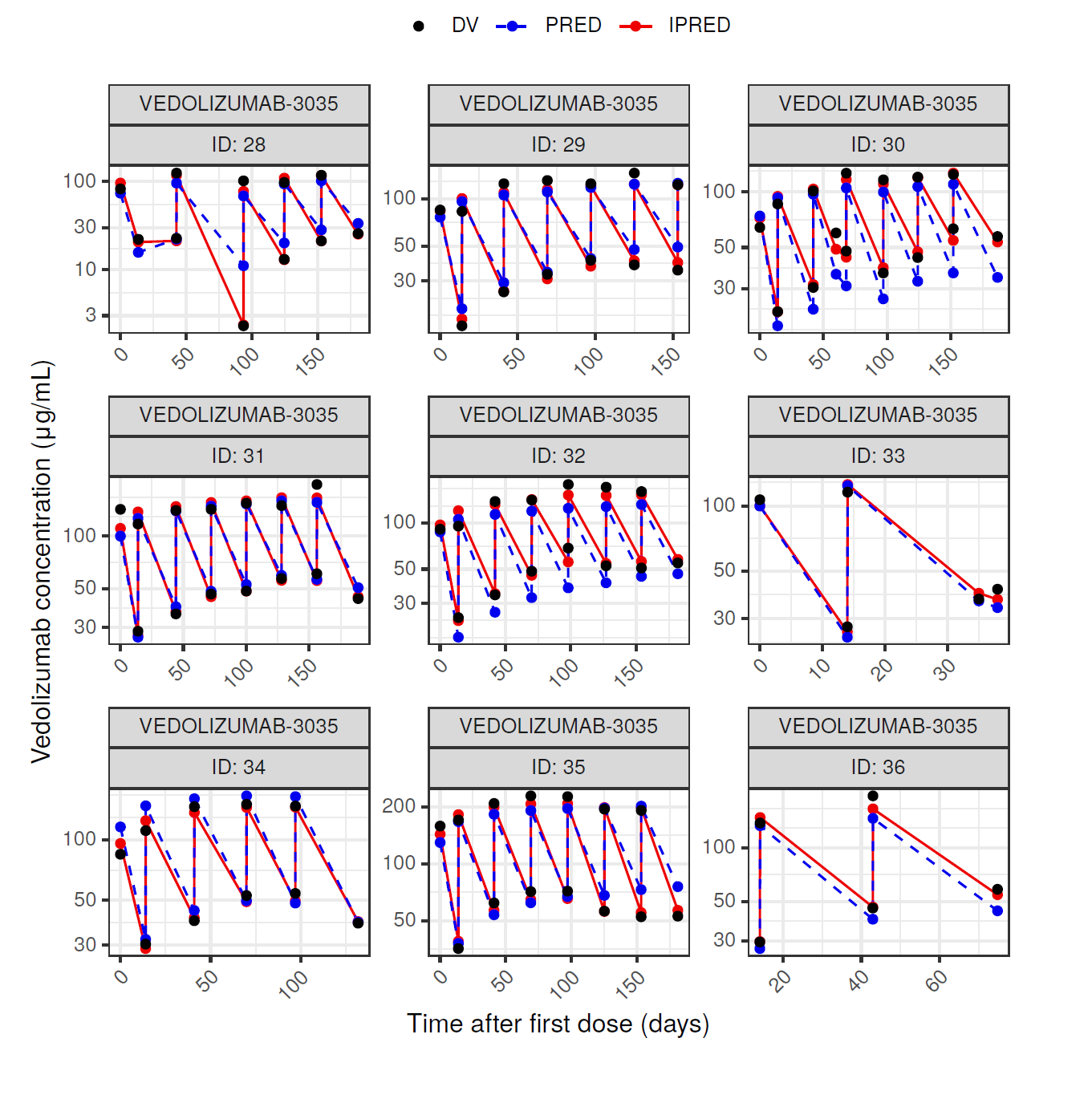


**Figure S8.** Final Model: Individual estimates of interindividual variability in vedolizumab pharmacokinetics by concomitant medication status.

# Figure S7. Vedolizumab NPDE versus predicted concentration and time after first dose for final vedolizumab model.

1. NPDE versus vedolizumab population predicted concentrations


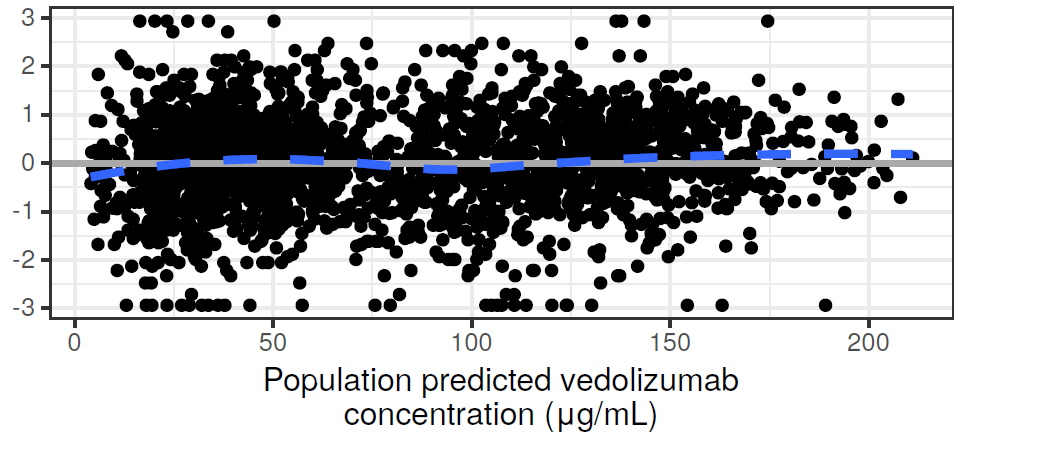


1. NPDE versus time after dose (days)


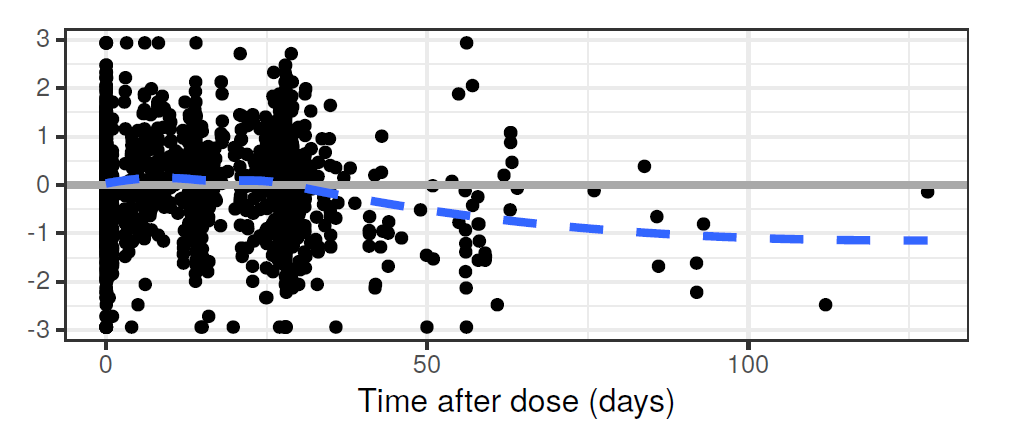


Values are indicated by black circles, the solid grey line at y = 0 is a reference line, and the dashed line is a LOWESS smooth trend line through the data.

NPDE, normalized prediction distribution error

# Figure S9. Vedolizumab NPDE versus continuous covariates for final vedolizumab model.

1. NPDE versus weight


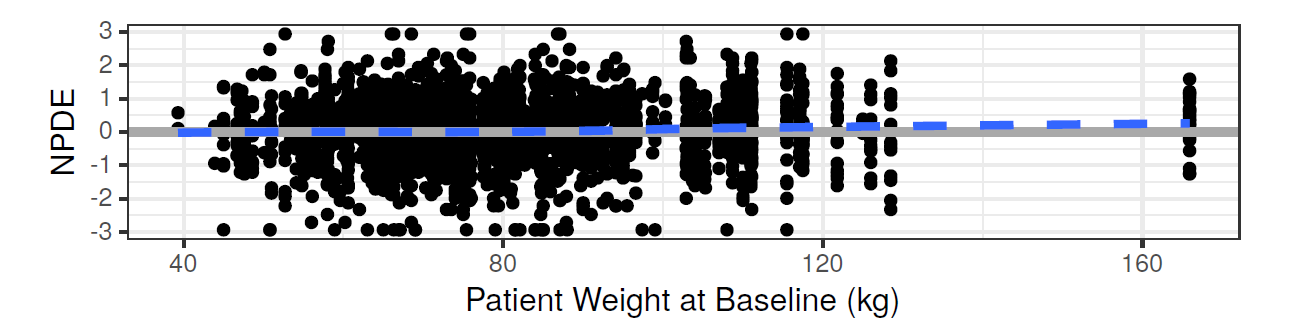


1. NPDE versus albumin


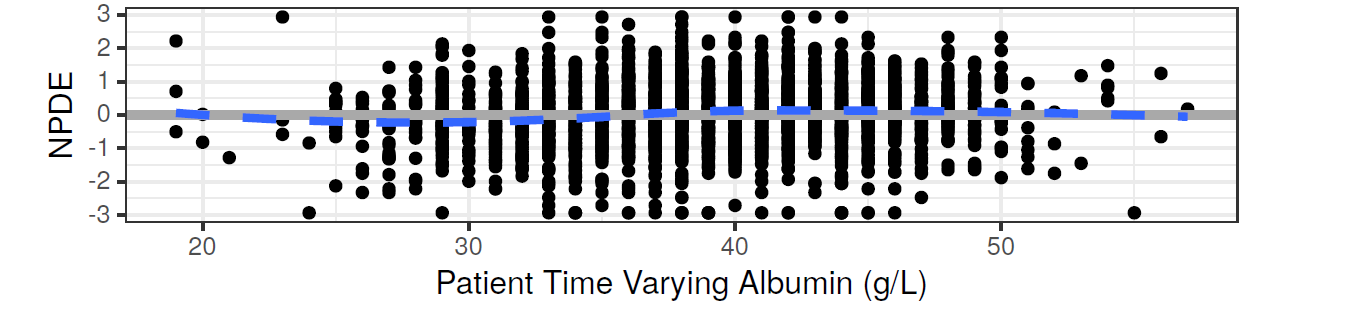


1. NPDE versus lymphocytes.


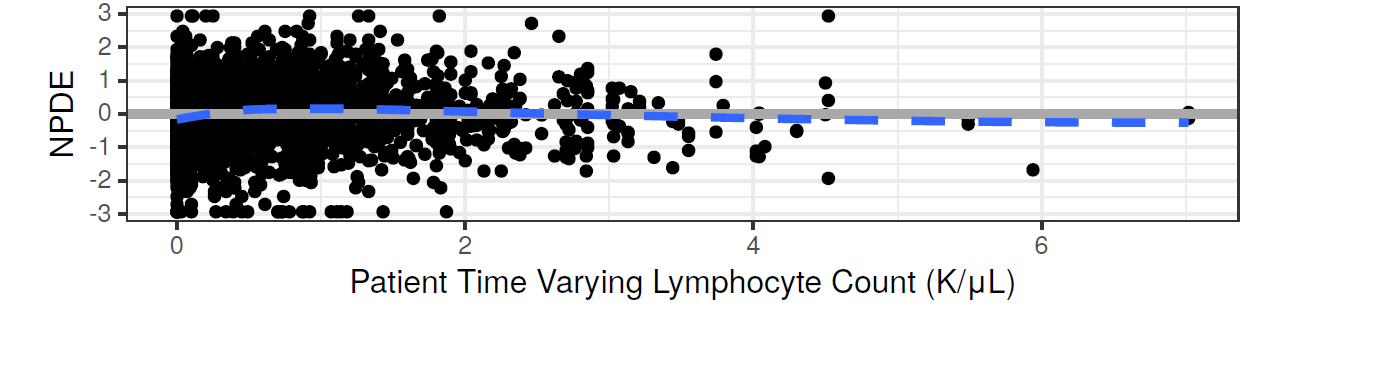


Values are indicated by black circles, the solid grey line at y = 0 is a reference line, and the dashed line is a LOWESS smooth trend line through the data.

NPDE, normalized prediction distribution error
